# Sensitivity of Different Developmental Stages and Resistance Risk Assessment of *Phytophthora capsici* to Fluopicolide in China

**DOI:** 10.3389/fmicb.2020.00185

**Published:** 2020-03-03

**Authors:** Jie Wu, Zhaolin Xue, Jianqiang Miao, Fan Zhang, Xiang Gao, Xili Liu

**Affiliations:** ^1^Department of Plant Pathology, College of Plant Protection, China Agricultural University, Beijing, China; ^2^Institute of Plant Protection, Hebei Academy of Agricultural and Forestry Sciences, IPM Center of Hebei Province, Baoding, China; ^3^State Key Laboratory of Crop Stress Biology for Arid Areas, College of Plant Protection, Northwest A&F University, Yangling, China

**Keywords:** *Phytophthora capsici*, fluopicolide, development stages, biological traits, resistance risk assessment

## Abstract

Sensitivities of *Phytophthora capsici* to fluopicolide were investigated *in vitro*, with results showing that fluopicolide had strong inhibitory activities on each development stage of *P. capsici*, in particular on the motility of the zoospore. The potential resistance risk for fluopicolide in *P. capsici* was evaluated. The baseline sensitivities to fluopicolide of 146 isolates obtained from 28 provinces in China were initially determined, and the 50% inhibition of mycelial growth (EC_50_) distribution was a unimodal curve with a mean of 0.17 μg/ml. A series of fluopicolide-resistant mutants of *P. capsici* were obtained by fungicide adaptation, and their biological traits were determined. Most of the resistant mutants showed similar favorable fitness in mycelial growth, sporangium and zoospore production, cystospore germination, and pathogenicity compared with their sensitive parents, with few exceptions. Additionally, the cross-resistance result indicated that the sensitivity of fluopicolide did not correlate with other oomycete fungicides, apart from fluopimomide (LH-2010A). These results suggest a moderate to high resistance risk of *P. capsici* to fluopicolide in China.

## Introduction

Oomycetes morphologically resemble fungi but are phylogenetically distant from true fungi (Baldauf et al., 2000; Tyler et al., 2006). *Phytophthora capsici* Leonian is a destructive plant oomycete pathogen that infects more than 70 species of plants, including most solanaceous and cucurbitaceous crops, causing crown, root, and fruit rot (Leonian, 1922; Erwin and Ribeiro, 1996; Brazier, 1998; Granke et al., 2012; Lamour et al., 2012). This can cause severe crop yield reduction and economic losses under suitable environmental conditions (Hausbeck and Lamour, 2004; Lamour et al., 2012). Billions of dollars from vegetable production are estimated to be threatened by *P. capsici* each year all over the world (Lamour et al., 2012).

Fungicides play the most important role in controlling *P. capsici*, combined with farming operations and biological control measures (Hwang and Kim, 1995; Hausbeck and Lamour, 2004). However, fungicides for the control of *Phytophthora* also present challenges because of the differences between oomycete and fungi, and resistance development (Lamour and Hausbeck, 2000; Lee et al., 2008; Randall et al., 2014). For instance, the resistance of *P. capsici* to phenylamide fungicides (e.g., metalaxyl or mefenoxam), used widely for many years around the world, has been reported universally (Luo et al., 1999; Lamour and Hausbeck, 2000; Parra and Ristaino, 2001; Silvar et al., 2006; Dunn et al., 2010; Quesada-Ocampo et al., 2011). Therefore, it is particularly important to assess the resistance risk and monitor the resistance development of oomycetes to available fungicides.

Fluopicolide is a benzamide plant fungicide discovered and patented by AgrEvo UK Limited (since 2002 being a part of Bayer Crop Science) in 1999. Its generic name is 2,6-dichloro-*N*-[[3-chloro-5-(trifluoromethyl)-2-pyridinyl]methyl] benzamide (C_14_H_8_Cl_3_F_3_N_2_O) (Lazzari et al., 2008; Zhang et al., 2019). Fluopicolide has been widely used for controlling a variety of oomycetes such as *Plasmopara viticola*, *Phytophthora infestans*, *Pseudoperonospora cubensis*, and *Bremia lactucae* (Lazzari et al., 2008; Rekanovic et al., 2008). It provides excellent efficacy on several pathogen developmental stages such as mycelial growth, sporangial production, zoospore release and motility, and cyst germination (Lazzari et al., 2008; Jackson et al., 2010). Studies have reported that the intracellular substances of hyphae or zoospores are leaked after fluopicolide treatment in *P. capsici* and *P. infestans*, and the potential target protein of fluopicolide is speculated to be a spectrin-like protein (Toquin et al., 2006; Jackson et al., 2010; FRAC, 2020), but this remains unclear.

The assessment of resistance risk for all novel pesticides has been required as part of the regulatory process in China since 2012 (NY/T1859.2-2012). The fungicide resistance risk assessment is essential to prevent or delay the development of fungicide resistance (Grimmer et al., 2014) and is mainly focused on the establishment of baseline sensitivity of sensitive pathogens, selection of resistant mutants, and evaluation of mutants’ resistance level, stability, and fitness, as well as the cross-resistance between the target fungicide and several others (Miao et al., 2016). At present, the resistance risk of *P. capsici* to fluopicolide in Michigan in the United States has been analyzed by Lu et al. (2011), and a few reports have described the sensitivity of *P. capsici* to fluopicolide in China using a limited number of isolates obtained from fewer than five provinces (Zhai et al., 2014; Wang et al., 2019). The objective of this study was to assess the resistance risk of *P. capsici* to fluopicolide in China in relation to the above aspects.

## Materials and Methods

### Pathogen Isolates, Plant Cultivars, and Culture Conditions

The total 146 *P. capsici* isolates were obtained from solanaceae and cucurbitaceae crops with typical brown lesions in 28 provinces of China during 2006–2014. The isolate A1 was obtained from Michigan and used for comparison with Chinese isolates. Isolates were cultured on a potato dextrose agar (PDA) medium in Petri dishes at 25°C in the dark for mycelial growth. Sporulation and zoospore production used the method reported previously (Miller, 1955; Lu et al., 2010). Isolates of *P. capsici* were cultured on V8 juice agar media plates (9-cm diameter) for 3 days in the dark at 25°C. Then, the plates were placed in a 12-h light/12-h dark photoperiod at 25°C for sporangial production. After 5 to 7 days, when sufficient numbers of sporangia had been produced, sporulating cultures were flooded with 10 ml of sterile distilled water and incubated at 4°C for 30 min, followed by 30 min at 25°C for releasing zoospores. The concentration of zoospores was measured and adjusted using a hemocytometer.

Pepper seeds (cv. Xichengdaniujiao) were sown in a seedling tray (540 mm × 280 mm × 50 mm) with a peat and vermiculite mixture (1:1 v/v) and a little chicken manure in a greenhouse (27°C ± 2°C, 80% relative humidity, and 12-h photoperiod). Pepper seedlings were cultivated to the six-true-leaf stage.

### Fungicides

Fluopicolide [97.2%, active ingredient (AI), Bayer Crop Science, Co., Ltd., Shanghai, China], zoxamide (97% AI, Gowan Company, United States), dimethomorph (95% AI, Gowan Company, United States), azoxystrobin (98% AI, Syngenta Biotechnology, Co., Ltd., Shanghai, China), fluazinam (95% AI, Japan Ishihara, Co., Ltd.), cyazofamid (98.4% AI, Mingde Lida Agricultural Technology, Co., Ltd., Beijing, China), metalaxyl (95% AI, Heben Technology, Co., Ltd., Zhejiang, China), chlorothalonil (98% AI, Henan Chunguang Agrochemical, Co., Ltd., Henan, China), oxathiapiprolin (96.7% AI, DuPont Crop Protection, Wilmington, DE, United States), and fluopimomide (LH-2010A, 98.6% AI, Shandong Joint Pesticide, Co., Ltd., Shandong, China) were respectively dissolved in dimethyl sulfoxide (DMSO) for stock solutions (10^4^ μg/ml) and stored at 4°C.

Cymoxanil (98% AI, Xinyi Agrochemical, Co., Ltd., Jiangsu, China) was dissolved in DMSO for stock solutions (10^5^ μg/ml) and stored at 4°C.

### Sensitivity of *P. capsici* to Fluopicolide *in vitro*

The sensitivity of *P. capsici* to fluopicolide in different developmental stages was determined by using dimethomorph as a comparative fungicide. The fungicide final concentrations are listed in Table 1, and the DMSO final concentration was adjusted to 0.1% (v/v). Each treatment consisted of three repetitions.

**TABLE 1:** Concentrations used to determine the sensitivity of *Phytophthora capsici* to fluopicolide and dimethomorph in different developmental stages.

Developmental stages	Fungicide concentration (μg/ml)	
	Fluopicolide	Dimethomorph
Mycelia growth	0, 0.08, 0.10, 0.13, 0.15, 0.20, 0.25, 0.30	0, 0.05, 0.1, 0.2, 0.4, 0.5
Sporangium formation	0, 0.025, 0.05, 0.1, 0.5, 1, 5	0, 0.005, 0.01, 0.05, 0.10, 0.20, 0.50
Zoospore release	0, 0.1, 0.5, 1, 2.5, 5	0, 1, 5, 10, 100
Cystospore germination	0, 0.5, 1, 2.5, 5, 10	0, 0.2, 0.5, 1, 2, 5
Zoospore motility	0, 0.01, 0.1, 1, 10	0, 1, 10

### Baseline Sensitivity of *Phytophthora capsici* to Fluopicolide

The sensitivity of the 146 *P. capsici* isolates (Supplementary Table S1) to fluopicolide was determined *in vitro* using the inhibition of the mycelia growth assay on PDA media amended with final concentrations of 0, 0.03, 0.10, 0.2, 0.4, and 1.20 μg/ml fluopicolide, and final DMSO concentration was 0.1% (v/v). Each concentration consisted of three replicate plates. The two perpendicular diameters (the 5-mm plug diameter was subtracted) of each colony were measured after 3 days at 25°C, and the effective concentration for 50% inhibition of mycelial growth (EC_50_) for each isolate was calculated (Pang et al., 2013).

### Selection of Fluopicolide-Resistant Mutants of *P. capsici*

The seven sensitive isolates (LP3, BYA5, HNJZ10, JA8, Pc1723, 12-11, and HD3) were obtained from Henan, Hebei, Gansu, and Jiangxi provinces of China. The isolate A1 was obtained from Michigan, and they were randomly selected from the laboratory’s *P. capsici* strains library. In pre-experiments, they showed good vitality at various developmental stages and were incubated on PDA plates for 3 days. Then, mycelial agar plugs (Φ = 5 mm) from the culture edge were placed mycelia-side down on PDA plates (Φ = 15 cm, 70 mycelia plugs per petri dish) amended with 5 μg/ml fluopicolide, approximately 10 times the minimal inhibited concentration of mycelial growth for sensitive isolates, which was tentatively considered to be a discriminatory concentration for identifying mutants of *P. capsici* insensitive to fluopicolide. After incubation at 25°C in the dark for 5 days, the faster-growing colonies were gradually transferred to new PDA plates amended with the same or higher fluopicolide concentrations (5, 10, 20, and 50 μg/ml). The colonies surviving on PDA containing fluopicolide were considered to be resistant mutants and were measured on PDA plates amended with a series of fluopicolide concentrations (0, 5, 10, 20, 50, and 100 μg/ml) to determine their resistance level. The number of these colonies was counted for each plate. This step was repeated until there was no significant difference in the linear growth of resistant colonies on the PDA plates with or without fluopicolide. Resistant colonies were transferred to new fungicide-free PDA plates for later tests. Mutation frequency was calculated as a ratio of resistant colonies to total number of colonies on plates.

### Characterization of Fluopicolide-Resistant Isolates

#### Resistance Factor and Stability

The mycelial growth of all *P. capsici* isolates was measured on PDA plates amended with two series of fluopicolide concentrations (0, 0.05, 0.1, 0.25, 0.5, and 1 μg/ml for wild-type sensitive isolates and 0, 5, 10, 20, 50, and 100 μg/ml for resistant isolates). A resistance factor (RF) was calculated as the ratio of EC_50_ values of a fungicide-resistant isolate relative to its parental isolate. The resistance stability of fluopicolide-resistant isolates was assessed after 10 successive transfers on fungicide-free PDA plates. The factor of sensitivity change (FSC) was calculated as the ratio of RF values of the 10th subculture relative to that of the first.

#### Effect of Temperature on Mycelial Growth

The fluopicolide-resistant isolates and their corresponding parental isolates were incubated on PDA media at 10, 18, 25, 30, and 37°C. Colony diameters were measured after incubation in the dark for 5 days, and each treatment consisted of three replicated plates.

#### Sporangium and Zoospore Production and Cystospore Germination *in vitro*

Sporangial production and zoospore release of mutants and parental isolates were measured as described above (Miller, 1955; Lu et al., 2010). Ten plugs (5 mm in diameter) from the culture edge and 10 from the area near the initial inoculum plug were placed into a 50-ml centrifuge tube containing 5 ml of sterile distilled water to produce zoospores. Sporangial production was assayed by counting the number of sporangia per square centimeter of V8 agar. Zoospore production was assayed by counting the number of cystospores in 200-μl suspension with a hemocytometer. Cystospore germination was assessed by plating cystospore suspension on the surface of 1% agar plates at 25°C in the dark after 12 h. More than 100 cystospores were examined under the microscope and considered germinated if the length of the germ tube was greater than the cystospore diameter. These experiments were conducted three times.

#### Virulence on Pepper Seedlings

Pepper seedlings were cultivated in the greenhouse as described above, and the inoculation of pepper seedlings and disease scoring were performed as in previous methods (Glosier et al., 2008), with minor modifications. The soil surface around each seedling was inoculated by adding 3 ml of zoospore suspension (2 × 10^4^ zoospores/ml) of resistant mutants or their parental isolates. Each treatment consisted of 20 seedlings of each isolate. The disease severity of all seedlings was rated after 7 days on a scale of 0–5: 0 = healthy plant, 1 = leaf yellowing and no stem necrosis, 2 = minor stem necrosis, 3 = moderate stem necrosis and some wilting, 4 = severe stem necrosis and severe wilting, and 5 = dead plant (Kim and Hwang, 1992; Hartman and Huang, 1993).

#### Cross-Resistance to Other Oomycete Fungicides

The sensitivities of 15 sensitive, seven intermediately fluopicolide-resistant, and eight highly fluopicolide-resistant isolates to 11 oomycete fungicides with different modes of action (Table 2) were determined by the mycelial growth inhibition method as described above. EC_50_ values were calculated as described earlier, and Spearman correlation analysis was carried out on log-EC_50_ values to test the sensitivity associations between fluopicolide and each of the other 10 oomycete fungicides. Each combination of isolate and concentration consisted of three replicate plates.

**TABLE 2:** Concentrations used to determine the sensitivities of fluopicolide-sensitive and fluopicolide-resistant *Phytophthora capsici isolates* to various fungicides.

Fungicide	Concentration (μg/ml)	
	For fluopicolide-sensitive isolates	For fluopicolide-resistant isolates
Fluopicolide	0, 0.05, 0.1, 0.25, 0.5, 1	0, 1, 2, 5, 10, 50
Dimethomorph	0, 0.1, 0.2, 0.3, 0.4, 0.5	0, 0.1, 0.2, 0.3, 0.4, 0.5
Zoxamide	0, 0.02, 0.04, 0.1, 0.4, 1	0, 0.02, 0.04, 0.1, 0.4, 1
Fluopimomide	0, 0.1, 0.5, 1, 2, 5, 10, 20	0, 0.1, 0.5, 1, 2, 5, 10, 20
Chlorothalonil	0, 0.2, 0.5, 1, 2, 5	0, 0.2, 0.5, 1, 2, 5
Cyazofamid	0, 0.1, 0.5, 1, 5, 10	0, 0.1, 0.5, 1, 5, 10
Azoxystrobin	0, 5, 10, 40, 80, 100	0, 5, 10, 40, 80, 100
Fluazinam	0, 0.5, 1, 5, 10, 25	0, 0.5, 1, 5, 10, 25
Metalaxyl	0, 0.2, 0.5, 1, 5, 10	0, 0.2, 0.5, 1, 5, 10
Cymoxanil	0, 20, 40, 80, 100, 150	0, 20, 40, 80, 100, 150
Oxathiapiprolin	0, 0.0002, 0.0004, 0.0006, 0.0008, 0.0015	0, 0.0002, 0.0004, 0.0006, 0.0008, 0.0015

### Statistical Analysis

All statistical analyses were conducted using DPS software ver. 7.05. The differences between the means of EC_50_ values were determined using Duncan’s multiple range test at *P* = 0.05. To plot the sensitivity of fluopicolide against those of 10 oomycete fungicides, EC_50_ values were transformed to relative logarithm values, and Spearman correlation analysis was conducted.

## Results

### Sensitivity of *P. capsici* to Fluopicolide *in vitro*

Fluopicolide was validated to have substantial activity against *P. capsici* in different developmental stages, with EC_50_ values of 0.09–1.58 μg/ml (Table 3). Compared to dimethomorph, fluopicolide had strong inhibitory activity on the mycelium growth *in vitro*. In particular, the inhibitory activity of fluopicolide on zoospore release was significantly better than that of dimethomorph. At concentrations as low as 0.01 μg/ml of fluopicolide, zoospores were swimming slowly; at concentrations of 0.1 and 1 μg/ml, 20–50% zoospores stopped swimming, then gradually swelled and burst in a few minutes, and the number of lysing zoospores increased with higher concentrations. At a concentration of 10 μg/ml, all zoospores lost motility and then gradually lysed after 10 min. However, all zoospores swam normally at the concentration of 1 and 10 μg/ml dimethomorph.

**TABLE 3:** Sensitivity of *Phytophthora capsici* to fluopicolide in different developmental stages.

Fungicide	Isolate	EC_50_ (μg/ml)			
		Mycelia growth	Sporangium formation	Zoospore release	Cystospore germination
Fluopicolide	LP3	0.28	0.12	0.16	1.20
	BYA5	0.18	0.09	0.30	1.58
Dimethomorph	LP3	0.30	0.03	>100	0.66
	BYA5	0.26	0.04	>100	0.52

### Baseline Sensitivity of *P. capsici* Isolates to Fluopicolide

**FIGURE 1:**
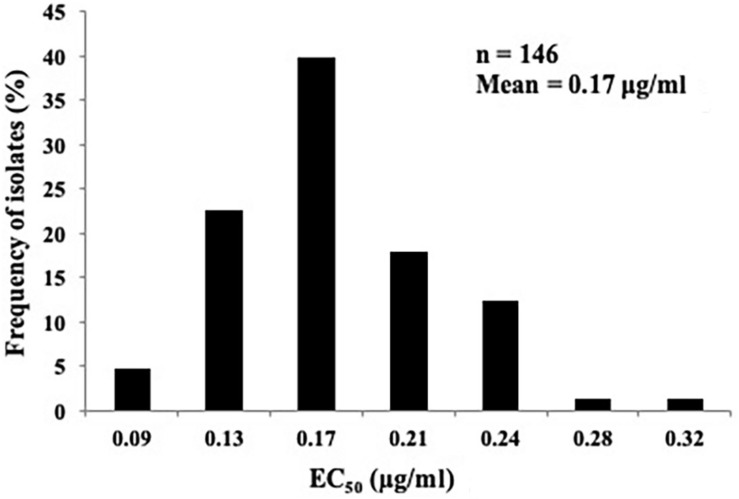
Distribution of fluopicolide sensitivity of 146 *Phytophthora capsici* isolates.

The sensitivity profile of *P. capsici* to fluopicolide was evaluated using 146 isolates collected from 28 provinces in China without a history of fluopicolide application (Supplementary Table S1). The individual fluopicolide EC_50_ values ranged from 0.07 to 0.34 μg/ml, and the frequency distribution of EC_50_ values was a unimodal curve, with a mean of 0.17 μg/ml (Figure 1). The narrow range and low EC_50_ values indicated that all wild-type isolates were sensitive to fluopicolide.

### Generation of *P. capsici* Mutants Resistant to Fluopicolide

Eight fluopicolide-sensitive parental isolates were exposed to fluopicolide for inducing and selecting mutants, and a total of 44 fluopicolide-resistant mutants were obtained from six parental isolates: LP3, BYA5, JA8, Pc1723, 12-11, and A1 (Supplementary Table S2). The mutation frequency, calculated as the number of resistant mutants divided by the total number of inoculations, was approximately 1 × 10^–4^. However, no resistant mutants were obtained from the other two parental isolates, HNJZ10 and HD3.

### Characterization of Fluopicolide-Resistant Mutants of *P. capsici*

#### Resistance Factor and Stability

The initial RFs of 44 mutants ranged from 6.21 to 559.90 (Supplementary Table S2). The resistance levels to fluopicolide were artificially considered as intermediate resistance if RF was less than 100 and high resistance if RF was more than 100 (Lu et al., 2011). According to this division, 30 intermediately resistant mutants and 14 highly resistant mutants made up the 44 fluopicolide-resistant mutants. After 10 transfers on fungicide-free PDA media, EC_50_ values of all mutants derived from parental isolates BYA5, LP3, 12-11, and highly resistant mutants of JA8 were increased. However, the EC_50_ values of intermediately resistant mutants derived from parental isolates JA8, Pc1723, and A1 decreased to some extent, except for RFJ-4 (Table 4). Overall, the FSC values showed relatively stable fluopicolide resistance of the mutants.

**TABLE 4:** Resistance stability of fluopicolide-resistant mutants of *Phytophthora capsici.*

Isolate	Origin	EC_50_ (μg/ml)		RF		FSC
		First	Tenth	First	Tenth	
BYA5	Parent	0.18	0.19	–	–	–
RFB-1	Mutant	3.90	6.85	21.82	37.00	1.70
RFB-4	Mutant	4.21	6.08	23.59	32.85	1.39
RFB-6	Mutant	3.18	6.57	17.80	35.48	1.99
RFB-9	Mutant	3.30	5.82	18.50	31.45	1.70
RFB-12	Mutant	8.31	11.11	46.50	60.01	1.29
RFB-3	Mutant	>100	>400	>559.90	>2,159.83	3.86
JA8	Parent	0.23	0.27	–	–	–
RFJ-1	Mutant	4.63	3.95	20.15	14.67	0.73
RFJ-4	Mutant	1.43	3.56	6.21	13.23	2.13
RFJ-8	Mutant	5.44	4.84	23.68	18.00	0.76
RFJ-13	Mutant	1.69	1.38	7.37	5.12	0.70
RFJ-17	Mutant	2.41	1.85	10.50	6.87	0.65
RFJ-7	Mutant	>100	>400	>435.45	>1,487.73	3.42
RFJ-9	Mutant	>100	>400	>435.45	>1,487.73	3.42
RFJ-12	Mutant	>100	>400	>435.45	>1,487.73	3.42
Pc1723	Parent	0.24	0.30	–	–	–
RF1723-1	Mutant	5.77	5.92	24.50	20.00	0.82
RF1723-6	Mutant	6.64	5.02	28.21	16.95	0.60
RF1723-7	Mutant	6.62	7.81	28.12	26.37	0.94
LP3	Parent	0.22	0.23	–	–	–
RFL-1	Mutant	>100	>400	>462.13	>1,722.70	3.73
RFL-2	Mutant	>100	>400	>462.13	>1,722.70	3.73
RFL-3	Mutant	>100	>400	>462.13	>1,722.70	3.73
RFL-4	Mutant	>100	>400	>462.13	>1,722.70	3.73
RFL-5	Mutant	>100	>400	>462.13	>1,722.70	3.73
RFL-6	Mutant	>100	>400	>462.13	>1,722.70	3.73
12-11	Parent	0.18	0.21	–	–	–
RF12-11-1	Mutant	>100	>400	>555.56	>1,900.11	3.42
RF12-11-2	Mutant	>100	>400	>555.56	>1,900.11	3.42
RF12-11-3	Mutant	>100	>400	>555.56	>1,900.11	3.42
RF12-11-5	Mutant	>100	>400	>555.56	>1,900.11	3.42
A1	Parent	0.23	0.42	–	–	–
RFA-1	Mutant	8.92	9.17	38.25	21.61	0.56

*EC_50_, 50% inhibition of mycelial growth; RF, resistance factor, ratio of EC_50_ of fungicide-resistant mutants relative to the EC_50_ of the parental isolate; FSC, factor of sensitivity change, the ratio of RF of the 10th transfer to the RF of the first transfer.*

#### Effect of Temperature on Mycelial Growth

The optimal temperature for mycelial growth of all tested mutants and their parents was validated at 25°C (Table 5). The mycelial growth rates of the mutants were significantly higher than those of their parents at 37°C, while some subtle differences in mycelial growth were observed among the mutants and the parents at the other temperatures. For example, resistant mutant RFB-3 grew faster than its parent BYA5, and RFL-2 grew more slowly than its parent LP3, at 10°C. The mutants derived from A1 and LP3, as well as mutants RFJ-4, RFJ-9, and RFJ-12, grew more slowly than their parents, and the hyphal growth rates of mutants RFB-1, RFJ-8, and RF1723-6 were higher than their parents at 18°C. The mutants derived from BYA5, Pc1723, 12-11, and mutant RFJ-8 presented faster mycelial growth, but LP3-mutants, RFJ-4 and RFJ-7 presented slower mycelial growth at 25°C. The hyphal growth rates of RFB-1, RF1723-1, and RFL-2 were not significantly different from those of their parental strains, and the hyphal growth rates of RFJ-4, RFJ-7, RFJ-9, RFJ-12, and RFL-3 were significantly slower than those of their parental strains, while the growth rate of other mutants was significantly faster, at 30°C. All resistant mutants grew faster than their parents at 37°C, which was speculated to be more conducive to survival in the summer.

**TABLE 5:** Effect of temperature on mycelial growth of *Phytophthora capsici* fluopicolide-resistant mutants and wild-type on potato dextrose agar plates.

Isolate	Origin	Colony grown diameter (mm)				
		10°C	18°C	25°C	30°C	37°C
BYA5	Parent	7.50 b	45.17 bc	54.17 c	45.67 b	0.00 b
RFB-1	Mutant	7.83 b	49.83 a	56.67 b	52.50 ab	1.00 a
RFB-12	Mutant	7.50 b	43.50 c	57.33 b	59.33 a	1.17 a
RFB-3	Mutant	10.17 a	46.50 b	59.50 a	56.00 a	1.50 a
JA8	Parent	8.17 a	44.33 b	57.33 b	51.50 b	0.00 e
RFJ-4	Mutant	7.67 a	38.33 c	50.00 d	23.50 e	1.17 de
RFJ-8	Mutant	7.33 a	47.67 a	64.50 a	56.00 a	16.17 a
RFJ-7	Mutant	7.33 a	43.00 b	54.00 c	45.50 c	2.17 cd
RFJ-9	Mutant	7.50 a	39.17 c	55.17 bc	42.50 c	3.50 c
RFJ-12	Mutant	8.17 a	39.33 c	57.00 b	36.50 d	6.67 b
Pc1723	Parent	7.67 a	44.33 b	52.17 c	47.17 b	0.00 b
RF1723-1	Mutant	8.00 a	44.00 b	54.83 b	47.83 b	6.00 a
RF1723-6	Mutant	8.83 a	46.33 a	64.17 a	65.83 a	5.67 a
LP3	Parent	6.67 a	50.00 a	55.33 a	48.83 a	0.33 b
RFL-2	Mutant	4.67 b	32.67 b	44.00 b	50.83 a	5.67 a
RFL-3	Mutant	6.00 a	22.83 c	27.33 c	23.50 b	3.67 a
12-11	Parent	8.00 a	41.17 a	51.00 b	35.17 c	0.30 b
RF12-11-3	Mutant	7.67 a	41.33 a	54.67 a	41.67 b	7.67 a
RF12-11-5	Mutant	8.17 a	41.67 a	55.17 a	50.00 a	8.67 a
A1	Parent	0.83 a	47.33 a	63.17 a	18.83 b	4.33 b
RFA-1	Mutant	1.00 a	44.17 b	60.50 a	45.00 a	13.00 a

*Different letters after the values indicate statistically significant differences (*P* < 0.05).*

#### Sporangium and Zoospore Production and Cystospore Germination *in vitro*

Sporulation of the fluopicolide-resistant mutants derived from BYA5, Pc1723, A1, and JA8 was greater than or similar to that of the corresponding parental isolates *in vitro*, while mutants derived from LP3 and 12-11 produced sporangia that were fewer or comparable to the parents. The zoospore release rates of the mutants were slightly higher or equivalent to the parents, except for RFB-12. No differences existed in zoospore production between resistant mutants and their corresponding sensitive parental isolates, except for mutant RFL-3, which produced significantly fewer zoospores than wild-type parent LP3. In addition, the sporangia malformation rates of mutants were significantly higher than those of the parents, where sporangia became significantly smaller and rounder. The cystospore germination rates of mutants obtained from BYA5, Pc1723, LP3, and JA8 were not significantly different from the parents, except for RFJ-4 and RFB-3, which produced significantly fewer germinated cystospores. In contrast, the cystospore germination rates of mutants obtained from A1 and 12–11 were significantly lower than those of the parents (Table 6).

**TABLE 6:** Fitness of *Phytophthora capsici* fluopicolide-resistant mutants and wild-type parents.

Isolate	No. sporangia (× 10^4^/cm^2^)	Malformation (%)	No. zoospores (× 10^4/^ml)	Cystospore germination (%)	Disease index
BYA5	483.60ab	2.35b	2.75a	92.50a	88.80a
RFB-1	437.40ab	1.98b	6.50a	77.50a	84.14a
RFB-12	327.00b	12.07a	3.75a	87.00a	83.70a
RFB-3	807.00a	4.90b	4.75a	40.93b	80.74a
JA8	601.80b	1.67b	5.25ab	87.5a	87.62a
RFJ-4	310.20b	21.52a	5.00ab	65.00b*c*	85.38a
RFJ-8	1,482.00a	1.21b	9.50a	92.50a	83.48a
RFJ-7	1,683.00a	1.14b	1.75b	85.00ab	71.54b
RFJ-9	618.00b	14.91a	6.75ab	95.00a	79.35ab
RFJ-12	975.00ab	2.56b	3.75b	95.00a	80.00ab
Pc1723	501.60a	9.57a	2.75a	87.83a	86.45a
RF1723-1	589.80a	12.62a	4.13a	60.00a	82.31a
RF1723-6	526.20a	17.17a	1.75a	65.00a	81.34a
LP3	521.40a	3.17c	1.75a	92.50a	80.35a
RFL-2	217.20b	62.71a	1.25ab	90.00a	86.67a
RFL-3	157.20b	40.66b	0.25b	85.00a	39.13b
12-11	1,746.00a	1.38b	5.00a	100.00a	84.00a
RF12-11-3	613.80b	6.39a	3.75a	70.00b	84.55a
RF12-11-5	1,075.20ab	6.97a	3.50a	77.50b	86.67a
A1	133.20a	21.47b	0.00a	100.00a	7.20a
RFA-1	133.20a	66.31a	0.00a	77.50b	2.61b

*Different letters after the values indicate statistically significant differences (*P* < 0.05).*

#### Virulence on Pepper Seedlings

The *in vivo* pathogenicity of resistant mutants was not significantly different from that of the parental isolates, other than RFL-3 and RFA-1, which had lower disease incidence and index (Table 6).

#### Cross-Resistance to Other Oomycete Fungicides

**FIGURE 2:**
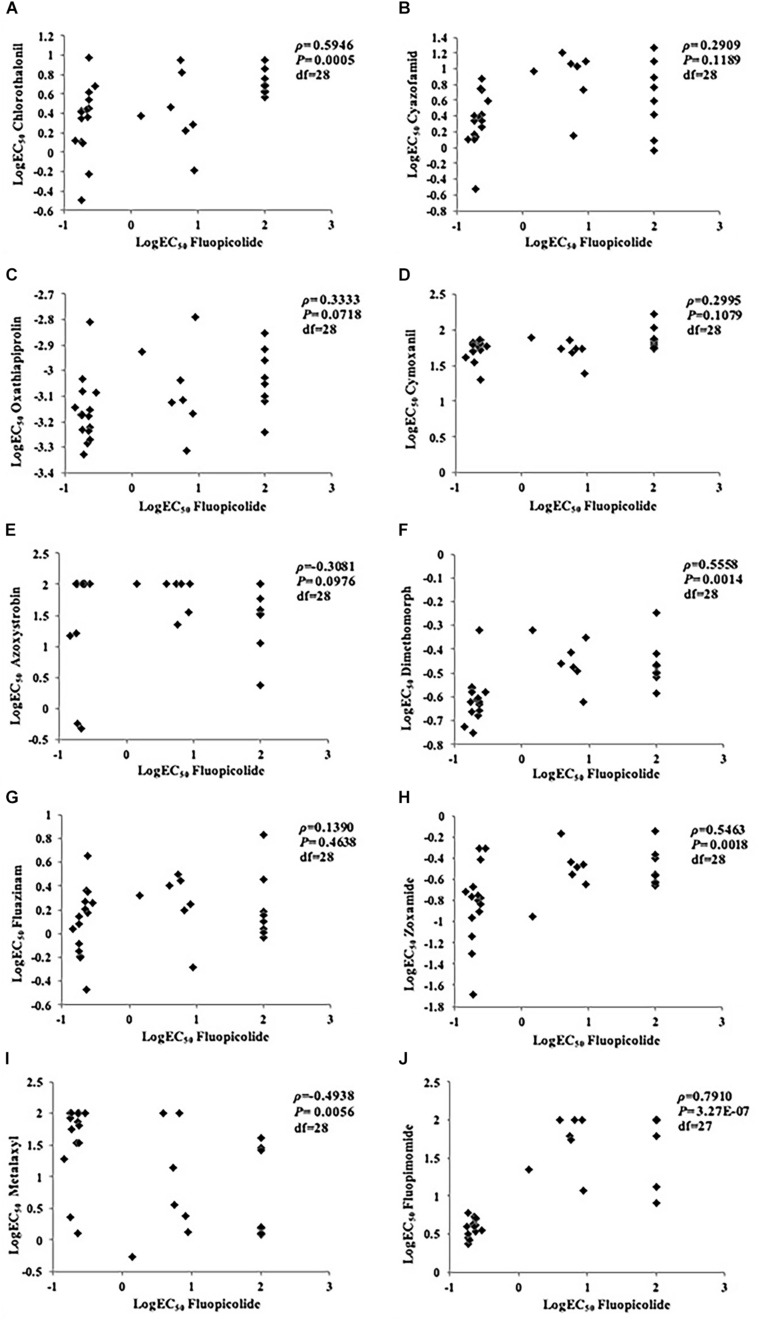
Rank correlation analysis for cross-resistance between fluopicolide and other oomycete fungicides in *Phytophthora capsici* isolates. **(A)** Chlorothalonil; **(B)** Cyazofamid; **(C)** Oxathiapiprolin; **(D)** Cymoxanil; **(E)** Azoxystrobin; **(F)** Dimethomorph; **(G)** Fluazinam; **(H)** Zoxamide; **(I)** Metalaxyl; **(J)** Fluopimomide.

No correlation was found between fluopicolide and the other five fungicides tested (cyazofamid, oxathiapiprolin, cymoxanil, azoxystrobin, and fluazinam), with *P*-values higher than 0.05 in rank correlation analysis for cross-resistance. Although a moderate correlation existed between fluopicolide and three fungicides (chlorothalonil, dimethomorph, and zoxamide) in rank correlation analysis, fluopicolide-resistant mutants were sensitive to these three fungicides, indicating that fluopicolide had no cross-resistance with them. In addition, positive cross-resistance was found between fluopicolide and fluopimomide (LH-2010A), contrary to metalaxyl (Figure 2).

## Discussion

This study determined the sensitivity of *P. capsici* to fluopicolide at different developmental stages. The results showed that fluopicolide had substantial activity against mycelial growth, sporangia formation, and zoospore release and motility, similar to previous studies (Lazzari et al., 2008; Jackson et al., 2010). In particular, zoospores stopped swimming, then gradually swelled and burst in a few minutes, at fluopicolide concentrations of 0.1, 1, and 10 μg/ml, compared to dimethomorph. This may be related to the suspected target of fluopicolide, a spectrin-like protein (Toquin et al., 2006), which may play an important role in maintaining the stability of the cell membrane. However, the specific mechanisms need to be further explored.

The fungicide resistance risk assessment is essential to monitor and manage the development of fungicide resistance in the field (Grimmer et al., 2014; Miao et al., 2016). The first step is to establish a baseline sensitivity for pathogen populations containing a large number of isolates (Russell, 2004). So far, only a few relatively comprehensive reports have described the baseline sensitivity of *P. capsici* to fluopicolide in the United States (Kousik and Keinath, 2007; Jackson et al., 2010; Lu et al., 2011). Lu et al. (2011) relatively comprehensively determined the baseline sensitivity to fluopicolide using 126 *P. capsici* Michigan isolates. However, due to geographical differences, this baseline sensitivity may not be suitable for assessing the resistance of *P. capsici* to fluopicolide in China. Furthermore, a few reports have focused on a limited number of isolates in very few provinces of China (Zhai et al., 2014; Wang et al., 2019). Zhai et al. (2014) merely determined the sensitivity of 42 *P. capsici* to fluopicolide from Tai’an, Pinggu, Hangzhou, and Kunming. Wang et al. (2019) only tested the sensitivity of some isolates of *P. capsici* in Chongqing. These limited strains and regions cannot comprehensively reflect the resistance situation in China as a whole and have less reference significance for resistance monitoring nationwide.

Therefore, this study has established a more comprehensive and accurate measurement of the baseline sensitivity of *P. capsici* to fluopicolide using 146 field isolates from 28 provinces throughout China from 2006 to 2014. Furthermore, the unimodal distribution of low EC_50_ values (with a mean of 0.17 μg/ml) has provided strong evidence that no fluopicolide-resistant subpopulations exist in the wild populations of *P. capsici* used in this study. Therefore, the current results based on a large number of isolates can be used to provide a baseline reference for monitoring resistance changes of *P. capsici* to fluopicolide in fields in China.

Fluopicolide-resistant mutants from mycelial plugs of wild-type isolates (LP3, BYA5, JA8, Pc1723, 12-11, and A1) were obtained by fungicide adaptation, which is representative of how resistance could develop naturally in fields. The resistance obtained by screening mycelial plugs on fungicide-amended media appeared with a relatively high frequency of approximately 10^–4^, which is much higher than that by screening zoospores, 10^–7^ (Lu et al., 2011). However, no resistant mutants were obtained from the other two parental isolates HNJZ10 and HD3, which might be due to sexual reproduction and genetic variation of those isolates (Hu et al., 2013; Pang et al., 2013; Miao et al., 2016). The resistance of mutants was stably inherited after 10 transfers of subculture on fluopicolide-free plates with stable RFs. The strains isolated from the inoculated pepper seedlings were still resistant to fluopicolide (data not shown).

Other biological characteristics of resistant isolates are also indispensable for evaluating fungicide resistance risks. Lu et al. (2011) previously determined the biological phenotypes of five resistant mutants and their corresponding five parents. In this study, the biological characteristics, which more fully reflect the biological viability of the mutants, of a total of 15 mutants with different resistance levels and six corresponding parental strains were determined. Studies of fitness showed that fluopicolide-resistant isolates exhibited strong adaptive traits in different developmental stages, including mycelial growth, sporangium production, cystospore germination, and pathogenicity. Notably, it was speculated that resistant mutants could be more conducive to survival in the summer as a result of faster growth than their parents at 37°C. This excellent adaptability indicates that the resistant mutants would be more competitive to infect crops successfully in fields and demonstrates that the subpopulations could successfully colonize, reproduce, and dominate in response to the selection pressure of fluopicolide. The superior ability of mutants to oversummer has not been reported before, which indicates that mutants can easily become dominant populations in the field, and the management of resistance cannot be ignored.

Although the resistance mechanism of fluopicolide needs to be further studied, the persistent resistance of fluopicolide-resistant mutants without fluopicolide selection pressure suggests that the resistance is a result of gene expression instead of acquired adaptation. Fluopicolide resistance was considered to be controlled monogenically and semidominantly because of the high RF values (>1,000) and spontaneous resistance development of resistant mutants (Lu et al., 2011). Therefore, fluopicolide may have a high inherent risk combined with the high activity and high fitness of resistant mutants. Due to the low inherent risk of *P. capsici*, a moderate to high resistance risk of *P. capsici* to fluopicolide is estimated in fields in China, according to the grading standards of Brent and Hollomon (2007). No positive cross-resistance exists between fluopicolide and other anti-oomycete fungicides, except for fluopimomide due to its similar structure. This suggests that fluopicolide should be applied limitedly in each season, or used alternately and mixed with other oomycete fungicides (except fluopimomide), to reduce or prevent rapid resistance development in fields.

## Conclusion

This study determined the sensitivity of *P. capsici* to fluopicolide and found that fluopicolide had substantial activity against mycelial growth, sporangia formation, and zoospore release and motility. The baseline sensitivity of *P. capsici* to fluopicolide has been established by using 146 field isolates from 28 provinces throughout China from 2006 to 2014, with a unimodal distribution of low EC_50_ values (with a mean of 0.17 μg/ml). Fluopicolide-resistant isolates obtained by fungicide adaptation exhibited strong adaptive traits in different developmental stages. No positive cross-resistance exists between fluopicolide and other anti-oomycete fungicides, except for fluopimomide. Therefore, a moderate to high resistance risk of *P. capsici* to fluopicolide is estimated in fields in China.

## Data Availability Statement

All datasets generated for this study are included in the article/Supplementary Material.

## Author Contributions

XL, JW, and ZX conceived and designed the experiments. JW, ZX, and JM performed the experiments. FZ and XG contributed reagents, materials, and analysis tools. XL supervised the work. ZX wrote the main manuscript. XL and JW revised the manuscript. All authors have read and approved the final manuscript.

## Conflict of Interest

The authors declare that the research was conducted in the absence of any commercial or financial relationships that could be construed as a potential conflict of interest.
