# Supplementary material for: Sensitivity of Different Developmental Stages and Resistance Risk Assessment of Phytophthora capsici to Fluopicolide in China
Source: Front Microbiol. 2020 Mar 3;11:185. doi: 10.3389/fmicb.2020.00185 (PMC7064020; doi:10.3389/fmicb.2020.00185)
Supplement: Supplementary file 1 [file Data_Sheet_1.docx]

Supplementary Material

**Supplementary Table S1.** ***P. capsici* isolates collected in China for study of baseline sensitivity to fluopicolide**

| Location | Host | Year | Number of isolates |
| --- | --- | --- | --- |
| Anhui | Pepper | 2006 | 5 |
| Beijing | Pepper | 2007 | 5 |
| Chongqing | Pepper | 2012 | 3 |
| Fujian | Pepper, Squash,  Cucumber, Eggplant | 2010, 2012, 2013 | 9 |
| Gansu | Pepper | 2007, 2011 | 15 |
| Guangdong | Pepper, Tomato | 2010, 2011, 2012, 2014 | 16 |
| Guangxi | Pepper | 2010, 2012 | 4 |
| Hainan | Pepper | 2009, 2011 | 4 |
| Hebei | Pepper | 2007 | 3 |
| Henan | Pepper | 2010 | 7 |
| Heilongjiang | Pepper | 2010, 2012 | 4 |
| Hubei | Pepper | 2009 | 3 |
| Hunan | Pepper | 2010 | 2 |
| Jilin | Pepper | 2012 | 3 |
| Jiangsu | Pepper | 2010 | 3 |
| Jiangxi | Pepper | 2012 | 6 |
| Liaoning | Pepper | 2012 | 2 |
| Neimenggu | Pepper | 2010 | 2 |
| Qinghai | Pepper | 2012 | 3 |
| Shandong | Pepper | 2010 | 3 |
| Shanxi | Pepper | 2007 | 3 |
| Sichuan | Pepper | 2010 | 8 |
| Taiwan | Pepper | 2009 | 2 |
| Tianjin | Pepper | 2009 | 3 |
| Xizang | Pepper | 2012 | 3 |
| Xinjiang | Pepper | 2009, 2010 | 2 |
| Yunnan | Pepper | 2010 | 20 |
| Zhejiang | Pepper | 2010 | 3 |
| Total | **--** | **--** | 146 |

**Supplementary Table S2. Resistance factors of fluopicolide-resistant isolates of *P. capsici***

| Isolate | Origin | EC_50_ (µg/mL) | Resistance factor (RF) |
| --- | --- | --- | --- |
| BYA5 | Parent | 0.18 | - |
| RFB-1 | Mutant | 3.90 | 21.82 |
| RFB-2 | Mutant | 3.66 | 20.51 |
| RFB-3 | Mutant | >100 | >559.90 |
| RFB-4 | Mutant | 4.21 | 23.59 |
| RFB-5 | Mutant | 3.66 | 20.51 |
| RFB-6 | Mutant | 3.18 | 17.80 |
| RFB-7 | Mutant | 2.92 | 16.35 |
| RFB-8 | Mutant | 3.71 | 20.76 |
| RFB-9 | Mutant | 3.30 | 18.50 |
| RFB-10 | Mutant | 4.14 | 23.16 |
| RFB-11 | Mutant | 3.28 | 18.38 |
| RFB-12 | Mutant | 8.31 | 46.50 |
| RFB-14 | Mutant | 3.15 | 17.65 |
| RFB-15 | Mutant | 3.25 | 18.18 |
| JA8 | Parent | 0.23 | - |
| RFJ-1 | Mutant | 4.63 | 20.15 |
| RFJ-2 | Mutant | 4.48 | 19.49 |
| RFJ-3 | Mutant | 4.70 | 20.47 |
| RFJ-4 | Mutant | 1.43 | 6.21 |
| RFJ-5 | Mutant | 3.86 | 16.79 |
| RFJ-6 | Mutant | 3.94 | 17.15 |
| RFJ-7 | Mutant | >100 | >435.45 |
| RFJ-8 | Mutant | 5.44 | 23.68 |
| RFJ-9 | Mutant | >100 | >435.45 |
| RFJ-10 | Mutant | >100 | >435.45 |
| RFJ-11 | Mutant | >100 | >435.45 |
| RFJ-12 | Mutant | >100 | >435.45 |
| RFJ-13 | Mutant | 1.69 | 7.37 |
| RFJ-14 | Mutant | 3.65 | 15.89 |
| Pc1723 | Parent | 0.24 | - |
| RF1723-1 | Mutant | 5.77 | 24.50 |
| RF1723-2 | Mutant | 3.67 | 15.58 |
| RF1723-3 | Mutant | 4.45 | 18.93 |
| RF1723-4 | Mutant | 6.26 | 26.60 |
| RF1723-5 | Mutant | 6.40 | 27.18 |
| RF1723-6 | Mutant | 6.64 | 28.21 |
| RF1723-7 | Mutant | 6.62 | 28.12 |
| LP3 | Parent | 0.22 | - |
| RFL-2 | Mutant | >100 | >462.13 |
| RFL-3 | Mutant | >100 | >462.13 |
| 12-11 | Parent | 0.18 | - |
| RF12-11-1 | Mutant | >100 | >555.56 |
| RF12-11-2 | Mutant | >100 | >555.56 |
| RF12-11-3 | Mutant | >100 | >555.56 |
| RF12-11-5 | Mutant | >100 | >555.56 |
| RF12-11-6 | Mutant | >100 | >555.56 |
| RF12-11-7 | Mutant | >100 | >555.56 |
| A1 | Parent | 0.23 | - |
| RFA-1 | Mutant | 8.92 | 38.25 |
